# Plant Polyphenols, More than Just Simple Natural Antioxidants: Oxidative Stress, Aging and Age-Related Diseases

**DOI:** 10.3390/medicines7050026

**Published:** 2020-05-09

**Authors:** Christophe Hano, Duangjai Tungmunnithum

**Affiliations:** 1 Laboratoire de Biologie des Ligneux et des Grandes Cultures (LBLGC), INRAE USC1328, Université d’Orléans, 21 rue de Loigny la Bataille, F-28000 Chartres, France; duangjai.tun@mahidol.ac.th; 2 Bioactifs et Cosmétiques, CNRS GDR3711, 45067 Orléans Cedex 2, France; 3 Department of Pharmaceutical Botany, Faculty of Pharmacy, Mahidol University, 447 Sri-Ayuthaya Road, Rajathevi, Bangkok 10400, Thailand

**Keywords:** aging, age-related diseases, antioxidant, coumarins, flavonoids, lignans, phenolic acids, polyphenols, stilbenes

## Abstract

The present editorial serves as an introduction to the Special Issue “Antioxidant and Anti-aging Action of Plant Polyphenols”. It also provides a summary of the polyphenols, their biological properties and possible functions as medicines, the importance of traditional medicines as a source of inspiration, the rationalization of new uses of plant extracts which lead to applications in modern medicine, the status of modern green-chemistry extraction methods, and some reflections on future prospects. Here, the articles from this Special Issue, and the main aspects of the antioxidant and anti-aging effects of plant polyphenols are discussed in the form of seven questions.

## 1. What Are These Polyphenols?

**Figure 1:**
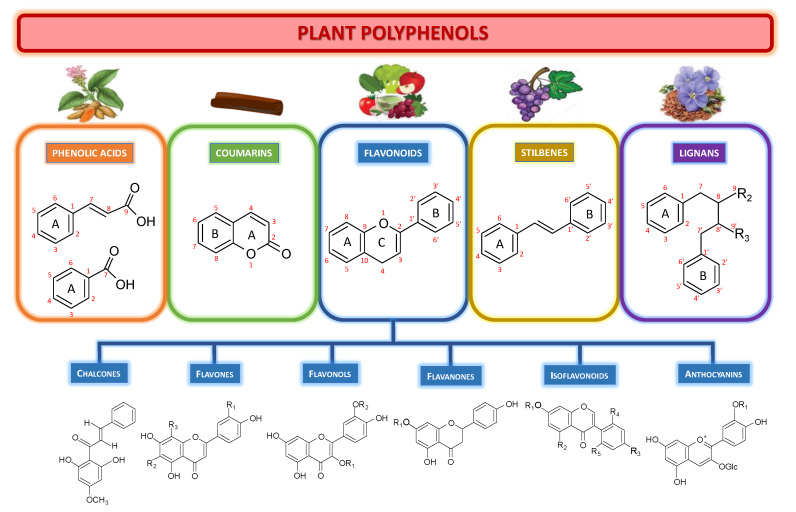
Polyphenol classification including phenolic acids, coumarins, flavonoids and their subgroups, stilbenes, and lignans.

Polyphenols are plant non-nutrient natural products or the so-called plant secondary metabolites found in fruits, vegetables and seeds that we consume daily. Polyphenols are a large family of compounds derived from secondary metabolism that are widespread in the plant kingdom. Most of these are derived from l-phenylalanine through the phenylpropanoid pathway. Sensu stricto, polyphenols are characterized by the presence of at least two phenolic groups associated in more or less complex structures, generally of high molecular weight, but simple phenolics (aka phenolic acids), that could be polyphenol precursors, are also considered to belong to this group. The most commonly used definition is: “The term “polyphenol” should be used to define compounds exclusively derived from the shikimate/phenylpropanoid and/or the polyketide pathway, featuring more than one phenolic unit and deprived of nitrogen-based functions” [1]. Polyphenols, therefore, include, but are not limited to, phenolic acids, coumarins, flavonoids, stilbenes and lignans (Figure 1). Other polymerized forms, such as tannins and lignins, are also included. Some of them are responsible for the aroma, color, antioxidant properties of the fruit, vegetables, seeds and nuts that we consumed. Polyphenols are becoming increasingly important, in particular because of their beneficial effects on health. Indeed, their role as natural antioxidants is increasing in the prevention and treatment of cancer [2,3,4], inflammatory, cardiovascular and neurodegenerative diseases [1]. Their intakes from fruit, vegetables, seeds, and nuts have been associated with lower risks of chronic and age-related degenerative diseases [5,6]. They have a wide range of applications as food supplements, pharmaceutical and cosmetic additives [1,2,3,4,5,6,7,8,9,10,11,12,13,14].

The various main classes of natural polyphenols are shown in Figure 1.

Phenolic acids (or phenolcarboxylic acids) are types of compound aromatic acids that contain an organic carboxylic acid function and a phenolic ring. Hydroxybenzoic acids (C6–C1 backbone), and hydroxycinnamic acids (C6–C3 backbone) are two essential forms of naturally occurring phenolic acids. These groups include benzoic acid, p-coumaric acid, but also more complex phenolic acids, such as curcumin from turmeric. 

Coumarins are benzopyrone (1,2-benzopyrones or 2H-1-benzopyran-2-ones) derivatives widely distributed in nature. Their name derives from the French word “coumarou” for the Tonka bean (*Dipteryx odorata*, Fabaceae), from which Vogel isolated coumarin in 1820). Chinese cinnamon is rich in coumarin derivatives.

Flavonoids are C6-C3-C6 phenylpropanoids consisting of two phenyl rings (rings A and B) and one heterocyclic ring (ring C). This group encompasses most antioxidants from fruits and vegetables, such as quercetin, kaempferol, isorhamnetin, fisetin, genistein. Flavonoids can be subdivided into different subgroups according to the carbon of the ring C connected to the ring B and the degree of unsaturation and oxidation of the ring C. Flavonoids in which the B ring is connected to position 3 of the C ring are called isoflavones. Those in which the B ring is associated in position 2 of the C ring can be further subdivided into several subgroups:

- Chalcones, characterized by the absence of ring C, therefore also called open-chain flavonoids.
- Flavones, with a double bond between positions 2 and 3 and a ketone on the ring C at position 4. Most of them have a hydroxy group in position 5 of the ring A, while hydroxylation in other positions, often in position 7 of the A ring or positions 3′ and 4′ of the B ring, are also commonly observed.
- Flavonols, the most common and the largest flavonoid subgroup, with a ketone and a hydroxyl group in position 3 of ring C. Flavonols exhibit various patterns of hydroxylation, methylation, and glycosylation. They can constitute the building blocks of proanthocyanin.
- Flavanones, with a fully saturated ring C. Unlike flavones, the double bond between positions 2 and 3 is saturated: this is the unique structural difference between these two subgroups.
- Anthocyanins, pigments responsible for the coloring of plants, flowers and fruits, which can vary depending on the methylation or acylation of the hydroxyl groups on the rings A and B but also the pH.

Stilbenes (aka stilbenoids) have a carbon backbone C6-C2-C6, namely the *trans*- ((*E*)-stilbenes) and *cis*-((*Z*)-stilbenes) 1,2-diphenylethylene structures. This group includes resveratrol from grape and wine.

Lignans are biphenolic compounds formed from the oxidative coupling of two monolignol (hydroxycinnamic alcohol) units. These same basic units are also used by plants to synthesize lignin, present in the walls of the conducting vessels. There are very many lignans, which differ in the type of bond between the two units and the changes that occur after dimerization. Secoisolariciresinol is one of the most common dietary lignans found in high amounts in flaxseeds.

## 2. How Can Simple Antioxidant Polyphenols Counteract Aging and Age-Related Diseases?

**Figure 2:**
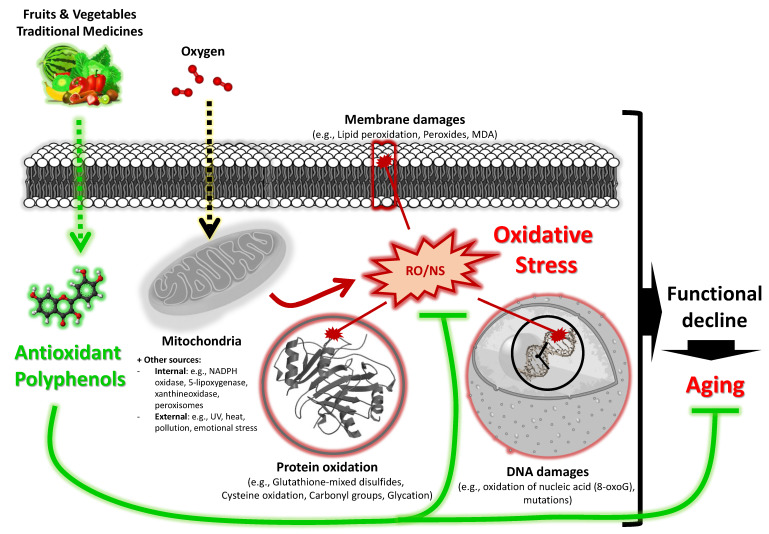
Schematic view of the premise behind the free radical theory of aging [16,17]. Mitochondria and other internal or external sources produced excessive oxidative stress (ROS/RNS) leading to oxidative damage to various cell macromolecules (membrane lipids, proteins and DNA) resulting in functional declines, aging and ultimately death.

Aging is a dynamic and complex biological process involving multiple actors and subject to a number of genetic and/or environmental influences [15]. A variety of theories were suggested to explain the aging process, including the free radical theory of aging proposed by Prof. Harman in 1956 [16]. Undoubtedly, this theory was the most widely studied and continues to be revised, and so far, it remains a sound theory [17]. The theory explains that aging can be caused by excessive oxidative stress (Figure 2) [17].

During oxidative phosphorylation, reactive oxygen and nitrogen species (ROS/RNS) are mainly formed within mitochondria, although these are generated by additional endogenous and exogenous factors. A network of endogenous and exogenous antioxidants neutralizes ROS/RNS, although some ROS/RNS still bypass these defensive systems. These escaped ROS/RNS subsequently cause oxidative damage to cellular components, including lipids, proteins, nucleic acids, etc. While mechanisms exist for repairing oxidatively damaged biomolecules, some damage remains. From this observation, the free radical aging theory assumes that ROS/RNS induce oxidative damage, causing cell dysfunction and physiological decline, leading to aging, with the appearance of degenerative diseases, and eventually death. This hypothesis therefore indicates that antioxidants which were successful in scavenging ROS/RNS are capable of slowing down the aging process. In line with this, studies have shown that different plant-derived antioxidants, in particular polyphenols, may have a therapeutic potential for aging and age-related diseases [18,19,20,21].

Evidence that polyphenols such as resveratrol and quercetin have prolonged the lifespan of different species, operating through a well-conserved mechanism, was first described in yeast and then confirmed in many other model species such as *Caenorhabditis elegans*, *Drosophila melanogaster* and mice [18,19,22]. Yeast cells prove to be an excellent model for evaluating the in vivo antioxidant capacity of polyphenols in the context of cellular oxidative stress [10,23,24,25,26,27,28]. It is also an attractive and stable eukaryotic model, whose mechanisms of defense and adaptation to oxidative stress are well established and can be extrapolated to human cells [23,24,25].

## 3. What Are the Polyphenols Used to Promote Human Health?

Beyond the simple antioxidant activity, the question of the health promotion action of polyphenols is a vast one. This point has been discussed in several papers in this Special Issue. In particular, in their review “Flavonoids and Other Phenolic Compounds from Medicinal Plants for Pharmaceutical and Medical Aspects: An Overview”, Tungmunnithum et al. [6] provide a comprehensive and synthetic description of the biological activities of plant polyphenols (flavonoids and phenolic compounds) in relation to their applied or potential pharmaceutical and medicinal potential. The authors present the effects of plant flavonoids and other phenolic substances on the promotion of human health, curing and prevention of diseases, including their antioxidant, antibacterial, anti-cancer, cardioprotective, immune system promoting, anti-inflammatory and skin protective actions. The natural plant phenolics and flavonoids with an interest in menopausal and postmenopausal women are also presented. Interestingly, the work of profiling and surveying flavonoids and other phenolics from medicinal plants is critically discussed, in particular, on the significant impact on the phytochemical quantity and composition of genetic (e.g., various ecotypes) and environmental factors, which represent a major challenge for the rationalization of traditional uses of medicinal plants, but also for their future use in modern medicine. Future perspectives and interesting directions for future research are also presented.

It is now recognized that the health-promoting effects of polyphenols are broader than their “basic” antioxidant function. The control of aging and degenerative diseases by polyphenols has also been linked to their ability to inhibit some enzymes such as cyclooxygenases and lipoxygenase 15 involved in inflammation [29] or acetylcholinesterase [30], associated with some neurodegenerative diseases in which oxidative stress and cholinergic deficiency create favorable conditions for Alzheimer’s disease or Parkinson’s disease. Minami et al. [31] describe an interesting action with Hochuekkito, a polyphenol-rich formula composed of 10 herbal medicines in traditional Kampo medicine, for the treatment of methicillin-resistant *Staphylococcus aureus* nasal colonization in the murine model, thus suggesting it as a serious therapeutic candidate for successful therapy in humans. Oxidative stress cross-talk between the host and *S. aureus* has been described as essential for nasal colonization [32,33]. It is therefore not excluded that polyphenols may interfere with this oxidative cross-talk.

## 4. To What Extent Can Traditional Medicines Be A Source of Inspiration?

The medical use of plants to treat, diagnose and prevent disease or maintain health is an important part of traditional medicine. Traditional medicines are an important source of inspiration for so-called modern medicine, which can contribute to the (re)discovery of lead remedies, as demonstrated by the success of antimalaria artemisinin and the Nobel Prize for the work of Prof. Tu Youyou. Beyond this well-deserved individual award, which comes to reward outstanding work, this award also highlights traditional medicines. We need to keep in mind that this is not an isolated case. Many plants have been used as an essential ingredient for various traditional medicines, such as traditional Chinese, Indian, Japanese, Thai, Korean, African, American or European medicines, and many new or unknown bioactive compounds have been discovered thanks to this ancient knowledge. In order to exemplify this traditional knowledge and how it continues to influence modern medicine through this special topic, a focus is placed on several plant species grown in various regions across the world, and used in various traditional medicines around the globe. 

Some papers focused on a single plant species, such as *Nelumbo nucifera*, widely used as an active component of traditional Chinese, Indian, Japanese, Thai and Korean medicines, and many others for a number of medicinal purposes, as shown in the review by Tungmunnithum et al. [34]. Le et al. [35] propose a green extraction of bioactive compounds from the fruits of Gac or *Momordica cochinchinensis* (Lour.) Spreng., a medicinally essential plant from northeastern Australia but also found in southern China, Thailand, Laos, Myanmar, Cambodia and Vietnam. 

Other papers pointed to the interest of several species of the same genus, allowing interesting cross-species comparisons, as reported by Koczka et al. [36] with *Rosa* species cultivated since ancient times, with a particular focus on *R. spinosissima*, *R. canina*, *R. rugosa*, *R. gallica* present throughout Europe, temperate Asia and North America, which have been used for their medicinal benefits for thousands of years. This is also the case with the work presented by Hennia et al. [37] on two *Myrtus* species, from the Mediterranean region (*M. communis* L., myrtle) and the Central Saharan Mountains (*M. nivellei* Batt. and Trab, Saharan myrtle)) used in folk medicines at the crossroads of different forms of traditional knowledge from both sides of the Mediterranean Basin. 

Also considered were different plant species from the same regions. Campaore et al. [29] presented work on two plant species from the Gampela region, located in the middle east of Kadiogo (central region, Burkina Faso), *Bidens engleri* (O.E. Schulz, *Asteraceae*) and the erect spiderling *Boerhavia erecta* (L., *Nyctaginaceae*), two well-known medicinal plants traditionally used in Burkina Faso and Cote d’Ivoire. Nwidu et al. [30] present a comprehensive ethnobotanical survey of plants from Niger Delta region (Nigeria) showing in particular the interest in *Musa paradisiaca*, *Dennettia tripetala*, *Moringa oleifera*, *Tetrapleura tetraptera*, *Terminalia catappa* and *Mangifera indica.*

Traditional medicine often used complex mixtures of various plant species in which the beneficial action resulted from synergies. As a result, the rationalization of the biological activity involved very challenging research in this situation. Minami et al. [31] present very interesting results on the biological evaluation (*in vitro* and animal model) of Hochuekkito, a formula composed 10 herbal medicines from traditional Kampo medicine. Minami et al. [31], in particular, show that if a single crude extract from *Astragali radix*, *Bupleuri radix*, *Zingiberis rhizoma*, and *Cimicifugae rhizome* is excluded from the Hochuekkito formula, the biological activity of the resulting new formula is significantly weakened.

## 5. How Can the Biological Activity of Conventional Plant Uses Be Rationalized Scientifically?

Scientific validation of the traditional uses of a medicinal plant is a crucial step before it becomes a lead drug, there are many applicants and few are chosen to consider the success of artemisinin as exceptional. Most of the traditional uses of medicinal plant species extracts need more research investigations before contributing to the discovery and large-scale production of potent drugs. Without being exhaustive, this Special Issue sheds light on a number of important considerations concerning the scientific rationalization of the biological activity of traditional medicinal plants. There may be several local names for the same plant, depending on the region and country, for the use of traditional medicines, and there may also be a similar name for another plant. Consequently, the authentication of a plant species is a critical issue to consider [34,37], and must be carried out before the biological evaluation or its use for medical and pharmaceutical applications. Authentication is a first step but several genetic and/or environmental factors may influence the phytochemical profile of a plant extract, and hence its biological activity [34,36]. The same observation can be made for extraction, and the conditions of extraction, in particular the selection of the extract solvent, can have a significant effect on the phytochemical composition of the extract and therefore on its activity [29,36]. The more complete phytochemical characterization of the extract is essential for achieving reproducible results, but our understanding of this composition is affected by the choice of the analytical method (UV-visible absorption, HPLC or GC coupled or not with mass spectrometry) and its resolution [34,37]. Identifying the bioactive compound(s) may be relevant, but sometimes the observed activity may be the result of complex synergism between different compounds from different plant extracts making it difficult to identify them [31]. Biological evaluation may be carried out in vitro as a first high-throughput screening, allowing the simultaneous evaluation of several extracts from different plants/conditions, prior to their evaluation *in cellulo* and/or with animal models, and prior to a more comprehensive toxicity assessment, clinical trials or epidemiological studies [29,30,31,34,35,36,37].

## 6. What Position Do the Modern Green-Chemistry Extraction Methods Have?

A number of methods for extracting natural antioxidants from various natural matrices have been developed. Conventional methods are based on maceration, infusion, and decoction but these are time-consuming processes. The use of plants is intended to return to more naturalness; hence the use of environmentally friendly extraction methods makes sense. More recently it has been shown that green extraction methods, including microwave-assisted extraction or ultrasound-assisted extraction, are especially successful. Le et al. [35] described the development of a green microwave-assisted extraction of bioactive compounds from *M. cochinchinensis* fruit. Such extraction methods have been shown to promote the increased solubility of compounds and the yields of extraction, as well as the reduction in extraction time and solvent consumption. When developing an extraction process, a key parameter to decide is solvent selection. Various solvents, including methanol, ethanol (EtOH), water or acetone, are used routinely for extraction of plant polyphenols but they are not all consistent with a green extraction process. Interestingly, water and ethanol, two of the most readily available solvents, are considered green solvents [36] and can be used in the development of green extraction methods [35]. Indeed, EtOH is one of the least toxic solvents for humans and more environmentally friendly than other organic solvents, such as methanol. In addition, the extraction capacity of EtOH can be easily modulated by adding water, making it an ideal solvent for the extraction of a wide variety of compounds with variable polarity [35]. These green extraction technologies have attracted a great deal of interest in industrial applications and are now considered to be one of the most efficient energy-saving processes in terms of length, selectivity and reproducibility.

## 7. Future Prospects: Does the Use of Plants in Modern Medicine Still Have A Future Today?

Most of the time, plants are readily available, cheap and relatively rich in polyphenols, which is why they were in the spotlight for traditional and alternative medicines as well as for research on health-promoting compounds. However, it must be borne in mind that this is a long way off. Complete knowledge of the phytochemical composition of a bioactive extract and its biological activity is important but not sufficient. Plant identification/authentication, harvesting and post-harvest treatment are important issues to consider. Genetics and/or the environment can have a significant impact on the phytochemical profile of the extract, affecting both its biological activity and its safety for the consumer. The rational identification of the bioactive compounds from the raw extract as well as the molecular targets of the compounds responsible for the activity are also important steps. The source plant, which does not necessarily produce a compound in sufficient quantities for industrial use, may be rare or endangered species. It is therefore sometimes necessary to design alternative methods of production. In addition to renewable sources, growing attention is being given to environmentally sustainable and consumer-friendly methods of extraction based on the principles of green chemistry. “Plant extract” or “natural product” does not inherently mean ‘safer’ than synthetic products in particular, as we still see so much in the minds of the general audience or the mass press, so toxicity and/or potential side effects need to be investigated. Targeted compounds should be used in biomedical and pharmaceutical research, ranging from in vitro to in vivo and clinical studies, to assess the safety, efficacy and side effects both short and long term of the candidate compounds tested. Despite all these obstacles before the discovery of an active molecule that will become a lead compound, polyphenols remain, and will certainly continue to be, serious potential candidates in the pharmaceutical and medical sectors to promote human health, prevent and cure various diseases. If we consider that only 15 per cent of the approximately 300,000 described terrestrial plant species have been systematically studied for their biological activities and/or phytochemical profiles, sometimes using ancient methods or not systematically and comprehensively, a vast field of exploration still appears to be open for the research on health-promoting polyphenols.
